# Getting an Early Start in Understanding Perinatal Asphyxia Impact on the Cardiovascular System

**DOI:** 10.3389/fped.2020.00068

**Published:** 2020-02-26

**Authors:** Mihaela Roxana Popescu, Anca Maria Panaitescu, Bogdan Pavel, Leon Zagrean, Gheorghe Peltecu, Ana-Maria Zagrean

**Affiliations:** ^1^Cardiology Department, Elias University Hospital, “Carol Davila” University of Medicine and Pharmacy, Bucharest, Romania; ^2^Department of Obstetrics and Gynecology, Filantropia Clinical Hospital, “Carol Davila” University of Medicine and Pharmacy, Bucharest, Romania; ^3^Division of Physiology and Neuroscience, Department of Functional Sciences, “Carol Davila” University of Medicine and Pharmacy, Bucharest, Romania; ^4^Intensive Care Department, Clinical Emergency Hospital of Plastic Surgery and Burns, Bucharest, Romania

**Keywords:** perinatal asphyxia, neonatal risk, cardiovascular, ischemia-reperfusion, myocardial development, prenatal programming, hypothermia, therapeutic approaches

## Abstract

Perinatal asphyxia (PA) is a burdening pathology with high short-term mortality and severe long-term consequences. Its incidence, reaching as high as 10 cases per 1000 live births in the less developed countries, prompts the need for better awareness and prevention of cases at risk, together with management by easily applicable protocols. PA acts first and foremost on the nervous tissue, but also on the heart, by hypoxia and subsequent ischemia-reperfusion injury. Myocardial development at birth is still incomplete and cannot adequately respond to this aggression. Cardiac dysfunction, including low ventricular output, bradycardia, and pulmonary hypertension, complicates the already compromised circulatory status of the newborn with PA. Multiorgan and especially cardiovascular failure seem to play a crucial role in the secondary phase of hypoxic-ischemic encephalopathy (HIE) and its high mortality rate. Hypothermia is an acceptable solution for HIE, but there is a fragile equilibrium between therapeutic gain and cardiovascular instability. A profound understanding of the underlying mechanisms of the nervous and cardiovascular systems and a close collaboration between the bench and bedside specialists in these domains is compulsory. More resources need to be directed toward the prevention of PA and the consecutive decrease of cardiovascular dysfunction. Not much can be done in case of an unexpected acute event that produces PA, where recognition and prompt delivery are the key factors for a positive clinical result. However, the situation is different for high-risk pregnancies or circumstances that make the fetus more vulnerable to asphyxia. Improving the outcome in these cases is possible through careful monitoring, identifying the high-risk pregnancies, and the implementation of novel prenatal strategies. Also, apart from adequately supporting the heart through the acute episode, there is a need for protocols for long-term cardiovascular follow-up. This will increase our recognition of any lasting myocardial damage and will enhance our perspective on the real impact of PA. The goal of this article is to review data on the cardiovascular consequences of PA, in the context of an immature cardiovascular system, discuss the potential contribution of cardiovascular impairment on short and long-term outcomes, and propose further directions of research in this field.

## Introduction

Asphyxia is characterized by hypoxia and hypercapnia that ensue due to lack of oxygen or ischemia. A limited degree of asphyxia is standard during childbirth (1). However, when prolonged or severe, fetal asphyxia may have harmful effects first and foremost on the fetal brain, but also other organs, like the heart, lungs, or the kidneys (2). Despite the efforts to reduce its incidence and consequences, perinatal asphyxia (PA) continues to occur globally in about 4 million babies every year (2) and accounts for 23% of all neonatal deaths and 8% of childhood deaths (3).

The incidence of PA depends on the definition used and the studied population. In developed countries, severe PA (defined by its consequences, death, or severe neurological impairment) is reported in 1 per 1,000 live births (4). In resource-poor countries, PA is more common and reflects the lack of access to adequate medical care. Reports from hospital-based studies in these settings suggest an incidence of 5–10 per 1,000 live births (5–7).

The main challenge for the clinician is the unpredictability of the phenomenon and the fact that once it starts, little can be done to minimize its harmful effects. Thus, it is easily understood why many preclinical and clinical studies have focused on finding early predictors for PA, as well as protective measures for both pre- and postpartum periods. The goal of the previously mentioned research is to generate standardized protocols that are readily available, especially in low-income areas, where PA is more prevalent.

Even if hypoxia leads to circulation redistribution to the brain, heart, kidney, and adrenal glands, a time-dependent injury still occurs in these organs. Although the majority of the studies have concentrated on the hypoxic brain injury, and how to diminish its impact, the effects on the cardiovascular system are equally important. Some studies report that 78% out of the total number of babies with severe PA suffered cardiac complications (2); in others, the value varies between 25 and 60% of cases (8, 9). The most extensive range is between 25 and 82% of the cases of neonatal asphyxia, comprising mild, moderate, and severe cardiac dysfunction (10–12).

Dysfunction of the cardiovascular system was repeatedly associated with a poor outcome in asphyxiated neonates (10, 13). Also, it seems probable that hypoxic-ischemic encephalopathy (HIE) is secondary to the multiorgan dysfunction, primarily to cardiorespiratory distress following PA, and not due only to poor oxygenation (14, 15). Hankins et al. (15) report that this was the case in 70% of babies in a clinical retrospective study. Thus, attention should be shifted away from the brain toward other organs that might consecutively impair the nervous system. New therapeutic targets could emerge by focusing on reestablishing normal circulation and counteracting the consequences of cardiovascular damage on HIE. Moreover, hypothermia, the primary therapeutic strategy currently in use for HIE (16, 17), has minimal positive effects on the heart and other organs.

This article aims to review the current data on the developmental status of the cardiomyocyte and cardiovascular system at birth and how it withstands neonatal asphyxia, the primary protective and treatment strategies currently being tested and to formulate possible future directions for research in this area. An additional target is to assist in early identification of the neonates at risk for severe cardiovascular dysfunction since this is the primary category that might benefit from rapid and efficient intervention and setting up innovative therapeutic protocols.

## Perinatal Asphyxia

Parturition is accompanied by a transient period of asphyxia (hypoxia and hypercapnia) in the neonate as part of birth stress, during the shift from maternal-fetal umbilical respiratory gas exchange toward fetal lungs activation. This non-pathologic condition can turn into prolonged perinatal asphyxia (PA) in birth complications like prolonged labor or umbilical occlusion, with long-term consequences, mostly on the highly hypoxia-sensitive immature brain (18).

Severe and prolonged asphyxia can result in either intrauterine fetal death and stillbirth or multiorgan failure in surviving neonates (2). Peripartum and intrapartum ischemic or hypoxic insults are, in many cases, impossible to anticipate and may be difficult to recognize before the delivery of the baby.

**Figure 1:**
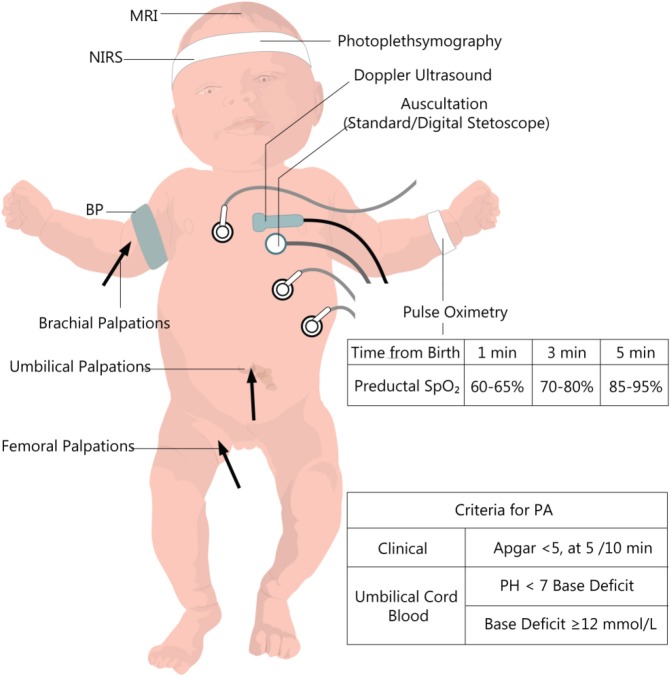
Clinical and paraclinical evaluation of the newborn with perinatal asphyxia. PA, perinatal asphyxia, BP, Blood pressure; NIRS, near-infrared spectrophotometry. Data from the European and American guideline recommendations for time-dependent preductal SpO_2_ (20, 21).

The most common neonatal signs of PA are described by the ACOG (American College of Obstetricians and Gynecologists) (19). They are presented in Figure 1, together with other clinical and biochemical measurements useful in the initial evaluation and further monitoring. These criteria only refer to those deliveries taking place at term or late preterm (gestational age [GA] ≥35 weeks). They outline essential principles to establish a causal link between intrapartum hypoxic events and long-term neurological outcomes. For babies delivered before 34–35 weeks' gestation, prematurity of their organs is likely to contribute to their long-term sequelae. The long-term post-asphyxia consequences depend on multiple factors such as gestational age, duration, and severity of the insult, pre-existent fetal pathology, and postnatal care. Also, maternal infections (22), nocturnal light exposure (23), behavioral hazards (e.g., nicotine and alcohol) (24, 25), diabetes (26), high-fat diet (27), or nutritional deficiencies (28) promote a more severe outcome than the actual degree of asphyxia.

Since a severe PA is commonly accompanied by a critical neurologic outcome, with HIE and its long-term devastating sequelae, most of the basic and clinical research in the field of PA has been focused on the brain (25, 29). In post-asphyxia HIE, brain lesions vary in severity, as classified by the Sarnat method (30) (stage 1–mild, stage 2–moderate, and stage 3–severe) which takes into account the baby's level of consciousness, neuromuscular and autonomic function, presence or absence of seizures and electroencephalographic recordings. This classification in stages of severity includes cardiovascular effects like irregularities of heart rate (HR) and blood pressure (BP), which are common during the period of reperfusion injury.

The mechanisms of post-asphyxia brain lesions imply the alteration of cerebral oxidative metabolism through a pathophysiological chain reaction, which ultimately leads to cellular death and brain damage. At the cellular level, brain injury after PA develops in two phases. The first phase occurs during and immediately after the hypoxic-ischemic insult and is characterized by global hypoxia. When the baby is delivered and resuscitated, but also during the first few hours of life, there may be an apparent stabilization. However, there is a second phase of the injury, which begins ~6 h after birth, characterized by post-hypoxic or post-ischemic hyperemia, with biochemical consequences such as excitotoxicity, oxidative stress, inflammation, and cytotoxicity (31).

However, there is still a lot to understand in the pathophysiological machinery underlying PA and to find an efficient treatment. The high level of unpredictability of PA makes the treatment strategies a real challenge. Finding novel solutions with regards to PA is a top priority for achieving the Millennium Development Goals set by the United Nations (7).

So far, the only available post-delivery option, along with gradual reoxygenation, remains exposing the child's brain to hypothermia to reduce the metabolic rate and the vascular permeability (32). Whole-body or head-only hypothermia started in the first 6 h after delivery, in selected neonates, reduces the neurological consequences of PA (33–35).

Hypothermia is thought to reduce oxidative stress, inflammation, and metabolic rate in the second phase of brain injury after PA. However, around 40% of neonates undergoing hypothermia still develop adverse neurologic outcomes. Many more that suffer PA are not eligible or do not have access to this therapy. For the treatment of brain hypoxic-ischemic encephalopathy, protocols of hypothermia (whole body or head only) are in place in almost all health care facilities in developed countries (36).

## Cardiovascular System and Perinatal Asphyxia

There is abundant data about the impact on the survival of cardiac dysfunction secondary to neonatal asphyxia (10, 37). Unfortunately, there are no follow up studies of long-term cardiovascular effects, neither for children with severe neonatal asphyxia with neurological sequelae nor for the ones with mild or moderate asphyxia episodes. The only cardiovascular pathology accompanying perinatal asphyxia with studies on long-term follow up is the persistent pulmonary hypertension of the newborn (38, 39).

In the following section, we will refer to the developmental features of the myocardium during the perinatal and postnatal period and pathophysiological, clinical, and molecular aspects of cardiovascular dysfunction in the acute setting of perinatal asphyxia.

### Normal Myocardial Development From Pre- to Postnatal Period

**Figure 2:**
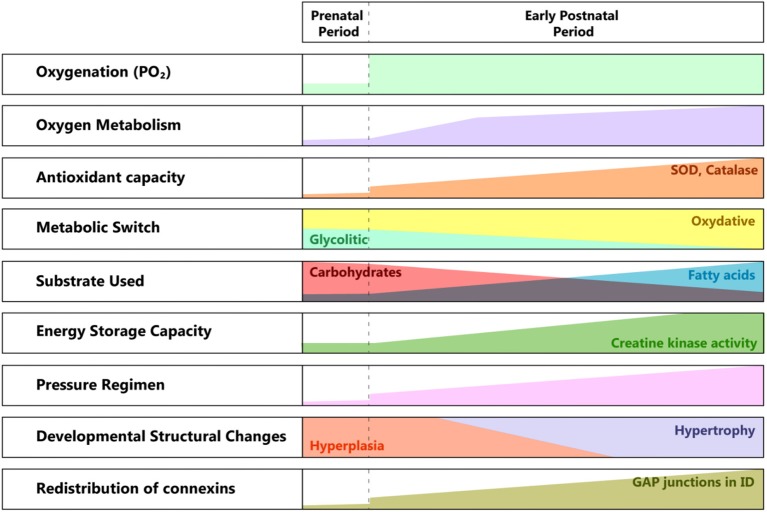
Developmental changes from pre to postnatal periods. SOD, superoxide dismutase; ID, intercalated disk; PO2, partial pressure of oxygen.

There are significant structural and metabolic differences between the fetal and the adult cardiomyocyte (40). The maturation process starts at birth when the partial pressure of oxygen increases and profound changes in the pressure regimen in the pulmonary and systemic circulation occur (see Figure 2).

The need for greater contractile force initially drives hyperplasia, followed by hypertrophy of the cardiac cell mass. The metabolic switch from glycolytic to almost entirely oxidative metabolism reflects the conversion of the substrate from lactic acid to fatty acids, in a newly oxygen-enriched environment (40).

The structural and ultrastructural changes are driven by the need for higher efficiency in energy production and better communication between the cardiomyocytes, in correlation with the higher functional requirements. The location of gap junctions varies between fetal and adult cardiomyocytes (41, 42), with an adult-like arrangement toward the end of the first year of life. The specific distribution at birth suggests a less well-coordinated syncytium than in the adult myocardium, hence its lower contractile force, especially under particular conditions (e.g., ischemia). This feature changes rapidly, as the connexins migrate toward the intercalated discs, transforming the cardiac cells in an efficient communicating mass.

Another important aspect is the vulnerability to the reactive oxygen species produced in the context of perinatal asphyxia and the ensuing ischemia/reperfusion lesion. Even if the antioxidant capacity accelerates during the last phase of gestation, at birth, it is still underdeveloped. There is a significant increase in superoxide dismutase (SOD) and catalase activity between the first and third months of extrauterine life. Also, there is a reduced capacity to react through the increased production of antioxidants (43).

### Physiopathology of the Cardiovascular Response to Perinatal Asphyxia

The neonatal myocyte survives in hypoxic conditions because of its mainly glycolytic metabolism. But, hypoxic conditions delay the transition toward more proficient energy production, from anaerobic to aerobic. The lack of metabolic switch leads to reduced ATP production, which in turn makes the myocyte's contraction less efficient. The internal cellular organization, not fully developed at this stage, further contributes to suboptimal excitation-contraction coupling. Also, the basal contractile state is higher than in adults, which impedes both diastolic and systolic function (44). The overall effect is of a barely surviving, less functional myocardial cell (45).

Moreover, during reoxygenation, hyperoxia can occur, and generate reactive oxygen species. The cardiomyocyte is still vulnerable to this new aggression, and its defense mechanisms are not fully developed yet. The adverse effects can range from organelle dysfunction to programmed cell death produced during reoxygenation and reperfusion.

The main physiological adaptation of the cardiovascular system to hypoxia is the redistribution of blood flow vital organs, mainly heart and brain (46). However, this is insufficient in cases of severe asphyxia, as both brain and myocardium develop ischemic lesions. In all other territories, vasoconstriction is translated into increased peripheral resistance. Hence, the moment of delivery is crucial, as, from a certain point, the high resistance in the fetal circulation short-circuits the blood to the low resistance placenta. The fetus remains with a small circulating blood volume. These facts may suggest the use of immediate clamping, but recent experimental data is contradictory (47). As Barberi et al. previously demonstrated, the newborn with severe asphyxia that correlates with signs of heart failure usually don't recover and die within the first week of life (10). This is another reason why efforts should be made to detect the severity of cardiac involvement as early as possible and focus on using all the available resources to stop or prevent further myocardial damage. Furthermore, apart from the low cardiac output that is due both to reduced contractile force and diminished preload, which is a consequence of pulmonary hypertension, neonatal asphyxia induces autonomic dysfunction, and vasoplegia (44).

The autonomic nervous system (ANS) is still incompletely developed at term. The sympathetic and parasympathetic evolve at different speeds during pregnancy, and this process continues for the following 6 months. Hence, it is essential to take the gestational age at birth into account when referring to autonomic dysfunction. Therefore, the more immature the autonomic system, the higher the disturbance in blood pressure and heart rate regulation will be.

Heart rate variability (HRV) is an excellent manner to investigate the well-being of the fetus, and it is also known to have prognostic significance (48). HRV is categorized as follows: (1) long term or low-frequency variability and (2) short term or high-frequency variability (49). Ultra-low frequency and very-low frequency HRV have been described, but they are under-evaluated in the newborn (48). Thus, we will only refer to high or low frequency HRV.

The long term HRV is considered by some authors to result from a combination of sympathetic, parasympathetic, and baroreceptor influences (50). Others believe it to be the product of the baroreflex function (51). In the case of the short term variability, the parasympathetic activity is thought to be responsible (52).

There is still debate over the afferent branch of the parasympathetic response. It appears that chemoreceptors are the main drivers of the cardiovascular response. There is no change in heart rate or blood pressure in hypoxic states when the carotid and aortic body are experimentally absent (53, 54). Also, an important aspect is the function of the baroreceptors, as their dysfunction makes the HRV an undependable substitute for blood pressure monitoring (55).

Some studies found connections between HRV, survival, and neurological outcomes in the newborn with HIE (56, 57). HRV was tracked during and after therapeutic hypothermia, showing a positive correlation between low HRV and adverse effects after birth hypoxia (56). This recommends HRV as a useful tool to complement EEG or even identify babies that would benefit from more aggressive adjuvant therapies, besides hypothermia.

HIE and its severity are linked to increased parasympathetic tone and lower sympathetic tone, seen as decreased HRV in the severely ill (58).

To date, it is unclear whether the ANS dysfunction itself contributes to secondary brain lesions, or, the depressed HRV, as a marker of ANS dysfunction, is just a biomarker of evolving brain injury in the context of perinatal asphyxia and emerging HIE.

### Cardiovascular System—Clinical Repercussions of Perinatal Asphyxia

Perinatal asphyxia triggers clinically relevant events within the cardiovascular system, ranging from heart rate, and systemic blood pressure variations to more profound changes, like myocardial dysfunction, myocardial ischemia, and pulmonary artery hypertension.

#### Persistent Pulmonary Hypertension of the Newborn (PPHN)

The transition from a high-resistance regime to low-resistance occurs in the pulmonary circulation in a very short time after birth. The adaptation is quite complex, involving the endothelium, a balance between mediators like endothelin 1, leukotrienes, and NO^60^. Hypoxia induces a disequilibrium in the ratio between endothelin and NO, in the favor of the former, causing vasoconstriction through endothelial dysfunction and decreased NO production. Also, if hypoxia is persistent, up-regulation of the NO synthase is blocked, and NO needed for pulmonary vasculature relaxation is lacking. This explains why treatment with inhaled NO is so efficient (59, 60). Later on, other added factors contribute to respiratory distress. These factors include left ventricular dysfunction, hypoxemia, oxygen administration, and even mechanical ventilation.

Persistent pulmonary hypertension of the newborn (PPHN) is associated with perinatal asphyxia. High vascular resistance is the effect of hypoxia on the pulmonary vasculature. Also, perinatal asphyxia is usually complicated by meconium aspirate and sepsis/pneumonia (12, 59, 61, 62). Most therapies to relieve pulmonary hypertension can have damaging effects on the cerebral circulation. Thus, special attention is needed in order not to do more harm than good. NO has proven a positive impact on the pulmonary circulation and the systemic arterial pressure (59). Venoarterial Extracorporeal Membrane Oxygenation (ECMO) is a lifesaving therapy in case of an oxygen index > 40 in spite of optimal NO treatment and mechanical ventilation (38). The oxygenation index represents: [FiO_2_/PaO_2_] × PAW where FiO_2_ is a fraction of inspired oxygen, PaO_2_ is the partial pressure of oxygen in arterial blood, and PAW is mean airway pressure. The survival rates for neonates on ECMO are over 80% but tend to decrease if the duration of this therapy lasts longer than seven days (63).

The recommendations for follow-up are similar to the ones for any pediatric pulmonary artery hypertension (39). PPHN is the only category of PA-related cardiovascular pathology that has the advantage of being included in follow-up protocols, thus being subject to consistent monitoring.

#### Myocardial Ischemia

**Figure 3:**
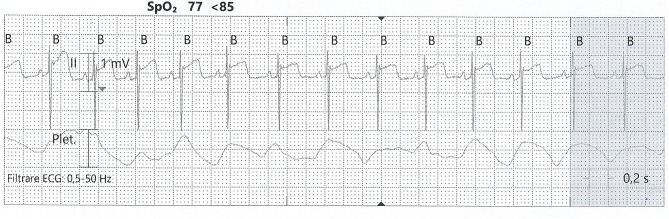
ST-elevation on an ECG tracing from a neonate with PA. SpO_2_, partial pressure of oxygen; Plet, plethysmography (from the collection of Neonatology Department, Filantropia Clinical Hospital, Bucharest, Romania).

As for any tissue, hypoxia leads to a mismatch between supply and demand, which results in decreased contractility (60). For adults, the protocol for recognizing hypoxic myocardial lesions is well-established. This is not the case for the newborn, where identifying ischemia is challenging. At birth, there is a lack of heterogeneity across the myocardial wall, concerning endocardial, midwall, and epicardial action potential (64). This may partly explain the repolarization aspect that resembles epicardial ischemia, with negative T waves. First attempts to identify cardiac injury in the neonatal setting analyzed only clinical signs and electrocardiography (ECG) tracings. In this manner, Martin-Ancel et al. identified 29% of patients with perinatal asphyxia to also have myocardial involvement (11). Barberi et al. stratified the severity of PA and observed the ECG patterns in these groups. The adverse outcomes correlate with the most severe ECG changes and also with the cardiac dysfunction documented through echocardiography (10) (see Figure 3). Troponin measurement is used in combination with CK and CK-MB for detecting cardiac injury.

#### Ventricular Dysfunction—Low Cardiac Output

The change from in parallel operation *in utero* of the two ventricles to in series operation *ex utero* implies profound changes in both metabolism and structure of the fetal myocardium converting to adult myocardium (40, 65). Unfortunately, this whole reorganization and function optimization of the heart, if superimposed on the metabolic challenge of PA, renders it unprepared to react and adapt appropriately. This perinatal insult may cause acquired neonatal cardiomyopathy in an otherwise structurally normal heart. The cardiac performance is affected, but this situation is usually reversible. Since this pathology requires early recognition and treatment, close cardiac monitoring of severe cases of PA is necessary.

As echocardiography emerged as a valuable diagnostic tool for bedside evaluation in the adult patient, it also started being investigated for systematic use in the neonatal setting (see section Evaluation of cardiac injury post-asphyxia). Assessment of systolic and diastolic function, as well as structural changes (e.g., ventricular dilation), is suitable for recognizing cardiac dysfunction and guiding therapeutic interventions. Furthermore, modern echocardiographic techniques, usually reserved for adults, are now being tested to validate their use in this very delicate field (66).

#### Arrhythmias

The best-documented arrhythmia during PA is sinus bradycardia, with a cut-off value of 80 beats per minute (14, 67). Below this value, the evolution is unfavorable, sometimes leading to terminal bradycardia (68, 69). Moreover, bradycardia lasting more than 13 min correlates with the development of cerebral palsy (68). Also, bradycardia is a critical time marker for delivery, as more than 18 min from uterine rupture until delivery leads to substantial morbidity (70).

Bradycardia occurs immediately in severe cases of perinatal asphyxia. It is a result of acute hypoxia and consequent acidosis (71). Vagotomy and atropine administration experimental studies demonstrated that the vagus nerve is responsible for the pronounced bradycardia seen in neonatal asphyxia (72–74).

Interestingly, the response to asphyxia is different *in utero* and after birth. The heart rate abruptly decreases if the umbilical cord is occluded while the fetus is still *in utero*, but the heart rate only mildly drops if the occlusion happens after delivery (75).

#### Hypotension

Blood pressure is initially preserved due to the redistribution of blood through peripheral vasoconstriction. In cases with continued asphyxia, when the fetus is apneic, blood pressure drops. The severity and persistence of hypotension will translate into the length of inotropic support and extensive fluid replacement (15, 76).

On the other hand, hypotension is also a consequence of bradycardia, knowing that neonatal cardiac output is significantly dependent on heart rate. Hypotension can occur either in the phase of secondary apnea, as previously mentioned, or as a side effect of therapeutic hypothermia (67, 77). Hypotension will gradually improve with the rewarming procedure (78).

### Perinatal Asphyxia Effect on the Neonatal Myocardium—Molecular Aspects

**Figure 4:**
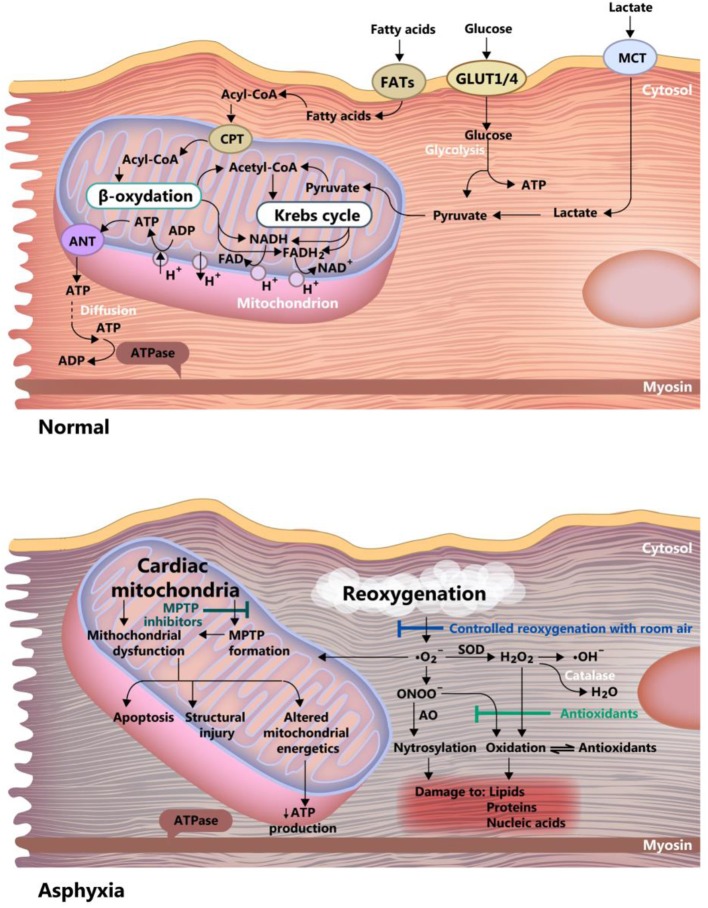
Differences between asphyxia and normoxia in the newborn myocardium cell. Acetyl-CoA, acetyl-coenzyme A; Acyl-CoA, acyl-coenzyme A; ANT, adenine nucleotide translocase; FAD and FADH_2_, oxidized and reduced, flavin adenine dinucleotide; FATs, fatty acid transporter; GLUT ¼, glucose transporter 1 and 4; MCT, monocarboxylate transporter; MPTP, mitochondrial permeability transition pore; ONOO, peroxynitrite; ATP, adenosine triphosphate; ADP, adenosine diphosphate; NAD and NADH, oxidized and reduced nicotinamide adenine dinucleotide, SOD, superoxide dismutase.

The healthy neonate myocardial cell is still adapted to work under relatively hypoxic conditions (i.e., 20–25 mmHg PO_2_), but with relatively low energy yield, using glycolytic pathways. This correlates with the fact that the heart does not need high performance until birth. In the case of perinatal asphyxia, this could prove to be beneficial, as the immature myocardial metabolism is adapted to low oxygen consumption, and fatty acid metabolism (adult) demands more oxygen than the glycolytic activity (40) (see Figure 4). Progressively, post-natal metabolism develops toward the adult cell organization and energy metabolism (see Figure 2). Thus, the neonatal cardiomyocyte is relatively resilient to ischemia, but as discussed below, less resistant to ischemia-reperfusion injury, mainly because of high vulnerability to the oxidative stress associated with reoxygenation.

The myocardial cell of the newborn is highly vulnerable to apoptosis (79), a feature that is further amplified by hypoxia through inhibition of adenosine triphosphate synthesis. The counteracting mechanisms in hypoxic-ischemic conditions are: (1) Controlled reoxygenation, using room air, to carefully titrate the oxygen saturation, a mentioned before. (2) Use of antioxidants and (3) Inhibition of the mitochondrial permeability transition pore. These mechanisms are graphically summarized in Figure 4.

**Figure 5:**
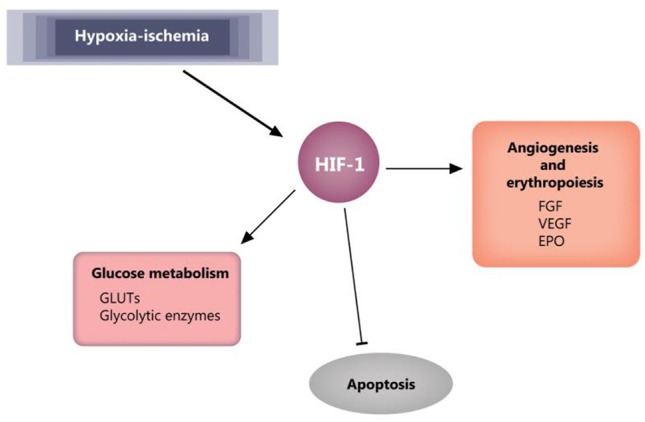
HIF-1 effects in ischemia. Blunt arrow indicate inhibition, pointed arrows indicate stimulation. EPO, erythropoietin; FGF, fibroblast growth factor; GLUTs, glucose transporters; HIF-1, hypoxia-inducible factor 1; VEGF, vascular endothelial growth factor.

A key player in cellular response to hypoxia/ischemia is hypoxia-inducible factor 1-α (HIF-1α), a transcriptional regulator of numerous genes involved in adaptive and survival mechanisms of cells transiting from normoxic to hypoxic conditions. Accumulation of HIF-1α impact on cellular metabolism by increasing the anaerobic glycolysis and diminishing mitochondrial oxygen consumption (80). Also, HIF-1α exerts protective effects by inducing vascular endothelial growth factor (VEGF) and erythropoietin (EPO) expression and supporting both angiogenesis and erythropoiesis (81). However, in the case of prolonged ischemia, HIF-1α can also have deleterious effects, like apoptosis and stunting cell growth (81, 82). Moreover, dependent on the gestational age at birth, HIF-1α was shown to have a role in signaling the mitochondrial transition toward an adult functioning pattern (83). The effects of HIF-1 are reviewed by Zhang et al. (81) and summarized in Figure 5.

Experimental studies have shown that cardiovascular phenotype can be modulated by environmental factors and pre- and perinatal events, like pre-eclampsia and perinatal hypoxia, through epigenetic transgenerational mechanisms (27, 84, 85). Epigenetic regulation of gene expression can result from DNA methylation/demethylation, histone modifications, and complementary binding of non-coding microRNAs (miRNA) to mRNA sequences. The fetal and neonate response to hypoxia is regulated by epigenetic mechanisms impacting on the synthesis and function of signaling molecules, receptors, ion channels, and enzymes, as recently reviewed by Ducsay et al. (80).

### Evaluation of Cardiac Injury Post-asphyxia

The cardiac activity and its structural integrity can be closely monitored using varied methods that have a long history of testing and validation. These include electrocardiography and heart rate variability, echocardiography, along with a biochemical assessment of diagnostic and severity markers.

Electrocardiogram was one of the first investigations used to diagnose myocardial injury. Barberi et al. use three groups (normal, mildly ill, and severely ill) of newborn babies to describe four stages of ECG changes, from T wave changes to ST elevation/depression (see Figure 3). These correlated with the severity of myocardial damage and also with the general status at birth (10). Grade 4 ECG changes were present only in the severe asphyxia group, and all of these babies died in the first week of life. Also, the myocardial function assessed by echocardiography showed a marked decrease only in the severe asphyxia group. Thus, ECG is a valuable tool in identifying myocardial damage, but it needs to be used in combination with echocardiography and biochemical markers.

Heart rate variability (HRV) serves to carefully follow the cardiac activity, altogether with the autonomic influence. Its prognostic value is described and recognized, in general, and lately also in neonatal care (86). A recent study describes the normal HRV pattern in the healthy newborn, even in the first 6 h, where the studies researching HIE are lacking information (48). Oliveira et al. describes in a recent review that all studies reporting HRV readings in HIE are consistent in predicting the neurological outcome of a baby suffering from PA (86). A decrease in HRV is associated with a poor outcome. For babies with PA, HRV analysis can assist in early risk stratification, and rush the decision for a particular therapeutic strategy, if performed promptly.

Echocardiography is a handy bedside tool for early diagnostic, functional cardiac evaluation, and monitoring. It is best performed as soon as there are signs of cardiovascular compromise. The neonatologist can assess the myocardial function, the presence of pulmonary hypertension, the persistence of shunting, and fluid repletion (10, 87–89). During therapeutic interventions, echocardiography can quickly depict changes in cardiac output, ventricular contractility or signs of pulmonary hypertension. Even more advanced techniques, like Tissue Doppler deformation indices of myocardial function, are currently used for neonate cardiac performance surveillance (90).

The biochemical markers are usually used in conjunction with clinical evaluation, ECG, and echocardiography. The most widely used are troponin T and I (77, 91–93), creatine kinase (CK), creatine kinase MB (CK-MB) (10, 13, 94), and the N terminal pro-brain natriuretic peptide (NT-proBNP) (95). There is a logical connection between low cardiac output, low coronary perfusion, and the release of troponin in the general circulation in asphyxiated infants (93). As in adult cardiology, there was an initial effervescence concerning troponin as a precise indicator of myocardial damage. Clinical experience shows that troponin tends to increase in many other clinical situations, as anemia, renal failure, tachyarrhythmia, or bradyarrhythmia. Still, it is the most widely used myocardial necrosis biomarker, as its dynamic is typical in myocardial infarction vs. other situations. CK and CK-MB are used together with troponin to depict myocardial damage. CK-MB rise has also been linked to renal impairment as part of the multiorgan disfunction associated with PA. Therefore, renal implication should be ruled out before confirming cardiomyocyte necrosis based only on CK-MB. NT-proBNP is used as a marker of adult and neonatal cardiac failure in general, and also for babies with PA (96). Also, Copeptin seems to be emerging as a potential marker of myocardial damage (95, 97).

### The Cardiac Injury Accompanying Perinatal Asphyxia- From Prevention to Treatment

**Figure 6:**
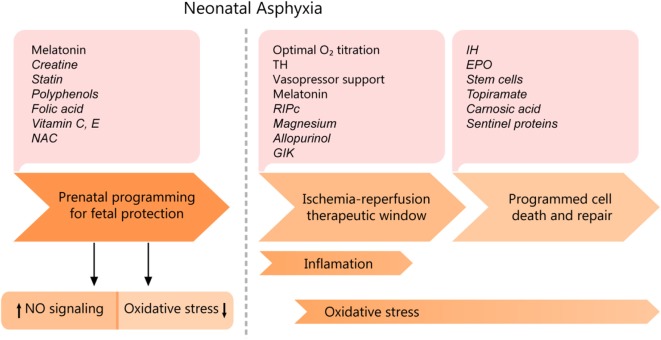
Cardiomyocyte asphyxic injury timeline and potential therapeutic interventions. EPO, erythropoietin; IH, Intermittent ischemia; GIK, glucose-insulin-potassium; NAC, N-Acetyl- cysteine; RIPc, remote ischemic pre/postconditioning; TH, therapeutic hypothermia. Experimental data are shown in italics.

Therapeutic strategies must be adapted to the timing of physio-pathological events and particularities of the cellular metabolism, as each evolving phase has a specific treatment for optimum results (see Figure 6). The proper conduct should be correlated with the severity of asphyxia, given the fact that mild asphyxia does not have such a drastic effect on the cardiovascular system. Only a few strategies are being currently employed, namely therapeutic hypothermia, decreasing the oxidative stress by carefully titrating the oxygen saturation, stabilizing the mitochondrial membrane, and supplementing the endogenous antioxidant capacity (43). However, an increasing amount of experimental data suggests high translational potential and will be discussed in the following sections, correlated with their mechanism of action.

#### Prenatal Programming

Treatment can start even before birth, as prenatal programming in pregnancies at high risk for perinatal asphyxia shows real promise. A few pathologies associated with high risk are maternal hypertension, preeclampsia, history of placental abruption, macrosomia.

##### Antioxidant therapy

Exogenous antioxidant therapy has proven positive effects both in experimental and human studies (98). The potential therapeutic value resides in the ability of different substances to reduce oxidative stress in the scenario of PA, given the fact that the myocardium newborn has diminished antioxidant defenses.

Melatonin, the pineal hormone exhibiting a circadian secretion with maximum nocturnal values, seems to have the best protective results, with its capacity to scavenge free radicals, reduce oxidative stress, and improve cellular physiology (99). In concert with its scavenging activity, melatonin at physiological levels was shown to increase the mRNA expression and *de novo* synthesis of glutathione peroxidase and superoxide dismutase (100). An essential aspect during gestation is the fact that maternal circadian rhythm impacts on the programming of fetal and newborn circadian clocks (101). The pineal gland develops during the 6–8th week postnatally, and the maternal melatonin is the sole melatonin source for the fetus and neonate (102). In this context, nocturnal light exposure, known as functional pinealectomy, acts as a potent circadian rhythm disruptor suppressing the endogenous circulating melatonin levels (103). Consecutive melatonin deficiency and circadian system disturbances in the fetus have shown an impaired reaction to stressors and long-term metabolic and behavioral consequences in the adult offspring (23). However, in the case of maternal administration of melatonin during late gestation, there is a reduced capacity of the newborn lambs to react suitably and to adapt neonatal asphyxia (104), possibly by an increased NO availability. These experimental observations recommend further research to establish the required melatonin dose and timing of administration during gestation, in agreement with concomitant risk factors.

A more recent experimental study associates melatonin with N-acetylcysteine, with positive results on antioxidant cardioprotection (105).

Other promising anti-oxidant substances in prenatal programming include folic acid, vitamin C, vitamin E, N acetylcysteine (NAC) (98), and polyphenols. One accessible dietary polyphenol with antioxidant properties is trans-resveratrol. When present in maternal diet, trans-resveratrol can diminish the PA-triggered inflammation and neuronal injury and increase the tolerance to asphyxia through transgenerational epigenetic mechanisms (85).

##### Statins

The effect of statins on endothelial progenitor cells (EPC) (106) mobilization, is relatively easy to study and implement, as there are ongoing studies for statin's effect on preeclampsia (107, 108). The downside is that the outcome will be difficult to evaluate, whether it will decrease the incidence of PA or increase the resilience to the hypoxic insult. To quantify these effects, large scale clinical studies are needed to observe the reduction in morbidity/mortality.

##### Creatine

Creatine is considered to be a worthy contender as a good source of energy in the context of PA. It can serve as energy supply not only for the brain but for the myocardium as well (2, 109). Multiple experimental studies using maternal diet supplementation with creatine show positive results, mainly on the diaphragm, but recent data indicate cardioprotective effects through HIF-1 activation (110).

#### Ischemia-Reperfusion Therapeutic Window

##### Reoxygenation with room air

Precautions must be taken in order not to cause more damage in the process of reoxygenation, by increasing the oxidative stress, through high production of reactive oxygen species. In the asphyxic newborn, the danger of hyperoxia is as serious as for hypoxia (111). Trying to correct the low saturation of oxygen rapidly can expose the neonatal myocardium to an oxidative stress level that is greater than its handling capacity. Current guidelines suggest following the physiological curve of oxygen saturation rise, as observed in healthy newborn (20, 21, 36).

##### Therapeutic hypothermia and its cardiovascular effects

The data about the impact of hypothermia on the cardiovascular system seems contradictory at first, with studies that confirm transitory adverse effects (14) and experimental data showing improved myocardial function after therapeutic cooling (8).

There was early controversy on hypothermia, as at first, it appeared to have a negative influence on the cardiovascular system. These adverse effects were sinus bradycardia, blood pressure decrease, and higher oxygen needs (9, 67). On the positive side, these effects were transient and subsided with the rewarming procedure.

These effects can be further categorized as affecting the heart rate or blood pressure (67). Regarding heart rate, there are accounts of arrhythmia, sinus bradycardia (<80 beats/min), prolonged QT interval, arrhythmia requiring cessation of the procedure, or immediate medical intervention. Where blood pressure is concerned, hypotension (mean arterial pressure below 40 mmHg), or the need for inotropic treatment was detected. Therapeutic hypothermia causes hemodynamic instability in 33–77% of neonates (78). Also, another occurrence was persistent pulmonary hypertension requiring NO inhalation (67).

Later reports showed better outcomes on myocardial damage with the use of therapeutic hypothermia (2, 67, 77). The myocardial injury was better assessed in these studies, both biochemically (CK-MB and troponin) and by echocardiography.

There are reports of a 67% reduction in cardiac output and a concordant decrease of heart rate and stroke volume during hypothermia, compared to the rewarming phase (112). A newer study found the opposite for stroke volume, in the context of decreased heart rate. They attributed the decrease in cardiac output during hypothermia to reduced metabolism, rather than a greater myocardial dysfunction (113). After rewarming, the myocardial function seems to be recovering faster than in non-cooled asphyxiated infants (90). Some studies ascribed this increase to a rise in heart rate rather than an increase in contractile force *per se* (76, 114).

##### Sustaining the circulation while the heart recovers

Multiple studies showed the beneficial effect of classical methods of resuscitation, comprising of fluid repletion, use of inotropic drugs, O_2_ delivery, and cardiopulmonary resuscitation in case of cardiorespiratory arrest.

It was believed at first that the higher the oxygen concentration, the better the outcome (115). This position was reconsidered because of the target organ ischemia/reperfusion lesions caused by brutal reoxygenation, as discussed earlier in this review and especially for the myocardium. The Resair study, on 609 asphyxiated newborn, helped with this position change (116). By 2005, even though the general conduct was to use room air vs. 100% oxygen, a Cochrane meta-analysis on five studies that included 1,300 infants, did not find enough compelling data to warranty the use of room air therapy as guideline initial treatment without a backup oxygen source (117). Both European and American resuscitation guidelines recommend using the preductal SpO_2_ compared with normal SpO_2_ from healthy newborns and time passed from delivery (see Figure 1) (36, 118). Even healthy newborn might have a lower saturation (<90%) for up to 14 min postpartum (111), so titration up to these values seems more adequate instead of a fixed FiO_2_ for the asphyxiated newborn. Also, there appears to be a need to individualize the treatment to avoid hyperoxia and at the same hypoxia in the more susceptible preterm male babies (119).

Apart from oxygenation, fluid resuscitation is another delicate subject, possibly causing cerebral edema if too aggressive (60). Unfortunately for the clinician, BP and perfusion are not always perfectly harmonized. Further evaluation, using modern techniques, has to be done to correlate blood pressure, cardiac output, and vascular resistance. Conventional parameters (plasma lactate, acid-base equilibrium, urine output) and newer methods [echocardiography and near-infrared spectroscopy—NIRS (120, 121)] have an essential contribution in relaying the whole picture. The advantage of the latter is the ability to provide functional data on repletion status (122) or myocardial function and also a real-time estimation of the tissue perfusion and oxygenation (120).

Accurately diagnosing the individual needs of the asphyxiated neonate will lead to the best decision in choosing the appropriate supportive therapy. The diagnosis concerns the myocardial function, the vasomotor function, and the pulmonary pressure. The ultimate purpose is to increase cardiac output and blood pressure and to decrease pulmonary pressure. The first line of treatment consisting of dopamine, dobutamine, epinephrine, and milrinone is completed by adjunctive therapy comprising norepinephrine, levosimendan, vasopressin, and hydrocortisone. This subject is extensively reviewed in a recent paper on supportive therapies of the asphyxiated newborn (44).

##### Melatonin

As previously mentioned, melatonin is extensively studied due to its potent antioxidant ability (99, 123). This capacity is of utmost interest in the context of perinatal asphyxia and the associated oxidative stress, as early postnatal treatment. Melatonin is neuroprotective in hypoxic-ischemic brain injury *in vitro* and *in vivo* animal models, and in asphyxiated term neonates when early administered post-asphyxia (124). Along with its antioxidant capacity, melatonin also elicits cardiovascular protection through anti-inflammatory, antilipidemic, and anti-hypertensive effects, which recommends it for clinical applicability (125).

Melatonin also acts as a protective molecule by favoring the parasympathetic system against the sympathetic one (126). This last effect is illustrated by a rise in plasma melatonin accompanying, as an adaptive mechanism, some cases of hypertension triggered by sympathetic overstimulation (127).

The cardiovascular effects of melatonin were recently described by Baltatu et al. in an elegant review of clinical and preclinical studies (128).

##### Ischemic preconditioning/post-conditioning

When perinatal asphyxia causes prolonged myocardial ischemia, the pattern of necrosis, apoptosis, infiltration with inflammatory cells, and scar formation can ensue. Histologic data confirming this come from experimental and necropsy studies (2). Ischemic preconditioning/postconditioning (129) emerged as a valuable method to use endogenous rescue mechanisms to decrease myocardial damage by ischemia/reperfusion injury (130, 131). Preconditioning has the advantage of having two phases, an early phase that occurs immediately and a late phase after 48–72 h after (132, 133). This biphasic pattern provides a longer window of protection. Even if these procedures are of practical use in severe neonatal asphyxia with a high risk of cardiovascular distress is still a question of debate. Intermittent hypoxia decreases the degree of apoptosis by reducing the endoplasmic reticulum stress (134, 135). There is some mechanistic similarity with remote ischemic preconditioning and also with pharmacological preconditioning (136). Remote preconditioning works for both brain (137) and myocardium (138, 139), but whether the same therapeutic window and dosages apply remains to be further documented. Furthermore, remote postconditioning seems to have positive results for both nervous tissue (140) and heart (94). The timely administration of these procedures could reduce the ischemia/reperfusion lesion in both the brain and myocardium, to reduce the morbidity/mortality of neonatal asphyxia.

##### Magnesium

Magnesium solutions are currently used intraoperatively because of its cardioprotective capacity during ischemia and reperfusion. This ability is linked to the blockage of L-type Calcium channels that produces a decrease of calcium loading, diminishing the myocardial energy demands (141) even in Langendorff-perfused hearts from young guinea pigs (142).

##### Other therapeutic strategies

Allopurinol and the noble gases such as *argon* (143) and *xenon* also show clinical promise but need further studies and confirmation, as some allopurinol results were contradictory to initial ones (144). Xenon is known as an ideal anesthetic agent with neuroprotective (145, 146) and cardioprotective properties (147). Its actions are mediated via KATP channels and other complex mechanisms converging toward antiapoptotic properties. In an international multicenter phase 3 study Hofland et al. proved that xenon anesthesia has cardioprotective effects on patients undergoing coronary bypass (148). Although xenon was used in neonates with brain injury and proved to be very safe but not superior to mild hypothermia (149), there are no studies to date that investigate xenon cardioprotective properties in neonates with cardiac failure following PA.

#### Cell Death and Repair

As the cardiac outcome is not the main target of research in neonates with perinatal asphyxia, studies into myocardial apoptosis and repair are lacking. We can only hope that experimental results will soon translate into practice.

##### Reducing the apoptotic process

Any mechanism reducing the apoptotic process is of particular interest in the context of myocardial ischemia/reperfusion injury. Selecting from the previously mentioned therapies, preconditioning, and melatonin appear to have the most convincing data. Intermittent hypoxia is beneficial, as it boosts the endogenous anti-apoptotic mechanisms (135). Melatonin inhibits apoptosis in mature cardiomyocytes (150), but also in H9C_2_ cells (151), which are similar to neonatal myocardial cells. Melatonin also regulates HIF expression (152). HIF-1 modulation plays a crucial role in regulating the apoptotic process (153). Glucose –insulin-potassium (GIK) solution, similar to the one used for adults to metabolically activate the myocardium, has a positive influence on the apoptotic process as well (154). So far, it was successfully used in a rat neonatal myocardium ischemia/reperfusion model (155), but not in human neonates.

##### Erythropoietin

Although consistent results in small rodents indicated erythropoietin as useful in reducing the myocardial infarction area and promoting cardiac repair (156), human studies didn't confirm these effects. The issue seems to be the timing of erythropoietin administration (157) and also with the adverse events.

##### Stem Cells

Stem cells research sprouted high hopes in regenerative medicine, with the perspective of progenitor cells replacing/improving the functionality of the ones damaged through hypoxic insult. Current data show the paracrine role of these cells and the stimulation of myocardial angiogenesis and repair (158). There are no follow-up protocols for cardiac dysfunction after perinatal asphyxia, apart from pulmonary hypertension; hence, stem cell protocols are hard to implement unless the cardiac dysfunction would be systematically sought after.

##### Other potential strategies

*Topiramate*, a sulfamate-substituted monosaccharide, a carbonic anhydrase inhibitor, similar to acetazolamide, was initially studied as an anticonvulsant and for neuronal repair and appeared to be useful in myocardial post-ischemia repair as well (159).

*Carnosic acid*, a phenolic diterpene with antioxidative properties, present in rosemary and common sage, was employed in ischemia-reperfusion injury and proved to be useful through the regulation of myocardial autophagy (160).

*Oxytocin*, a hypothalamic neuropeptide involved in maternal-fetal interaction, with neuroprotective roles during birth (29), is also released from peripheric tissues, including heart and endothelial cells. Oxytocin was experimentally studied for its cardioprotective effect and is currently examined for its potential use in clinical trials, as recently reviewed by Wang et al. (161). In the immature neonate myocardium in rat, oxytocin translocates glucose transporter 4 (GLUT4) to the plasma membrane, regulating glucose uptake, thus sustaining cell metabolism and myocardial function during hypoxia-ischemia. Oxytocin effects on GLUT4 were also found in human endothelial cells (162). Furthermore, oxytocin was shown to cooperate with atrial natriuretic peptide by acting as a natriuretic agent (163).

*Sentinel proteins*, such as poly(ADP-ribose) polymerase 1 (PARP-1), are activated in the context of oxidative stress to maintain genome integrity. Even if they have an initial beneficial effect, in the end, in the case of overproduction, they can cause cell death (164). Learning a lesson from the neuroprotective strategies, inhibition of sentinel proteins (165, 166) might be of assistance to the heart as well (167).

## Discussion of Future Directions

After extensive research of the literature, we did not find long-term follow-up studies of the children that suffered a mild or moderate degree of PA and not even of those that survived a severe episode with neurological sequelae. The only type of follow up is set up for cases with persistent pulmonary hypertension. But, a recent long-term experimental study shows ultrastructure and contractility changes in adult rat myocardium (168). Thus, discovering if there is a link between the initial severity of PA, the neurodevelopmental outcome and the cardiovascular status in adulthood would be of particular interest. Therefore, periodical check-ups of the cardiac function in all children with PA comprising physical examination, echocardiography, and ECG Holter monitoring, would undoubtedly improve our perception of the real effect of PA on the cardiovascular system.

The best way to prevent prenatal asphyxia is to closely monitor the fetus in a way that would prompt rapid delivery in case of an acute event (169). Better prevention can also be attained by training obstetricians to expertly interpret cardiotocographies (CTGs) (170). Every case needs to be taken separately to prevent PA in babies that are not at risk for a pathological event at birth, but they manifest nonetheless as PA. For some of these cases, the pregnancy should have been considered at risk and thoroughly investigated, for example, in diabetic or hypertensive mothers.

Thus, antenatal screening of mothers with hypertension, preeclampsia, markers of preeclampsia (PlGF/sFLT), etc. is an excellent way to identify pregnancies that would benefit from antenatal treatment, given the risk of PA. As discussed in section creatine (109) is such a substance, with the advantage easy access and without significant adverse effects.

Also, gestational exposure to potential deleterious factors (macrosomia, IUGR, ethanol, nocturnal light exposure) induces a lower offspring tolerance to milder asphyxia. Therefore, a child that was supposed to have normal parameters at birth has the typical characteristics of PA. Special precautions need to be taken in preventing this outcome by closely monitoring the pregnancies at risk and possibly administering prenatal therapies (e.g., melatonin), or recommending epigenetic diets enriched in bioactive compounds (e.g., resveratrol).

In general, adult treatment of cardiac dysfunction can be applied in neonates, although some of the methods are not yet thoroughly tested. As an example, the GIK solution (glucose-insulin-potassium) is used in treating cardiac dysfunction after cardiac surgery or myocardial infarction, with good long-term outcomes (154, 171, 172). However, GIK solution hasn't been tested in neonate cardiac dysfunction induced by the PA. There is one experimental study performed in neonatal rats, investigating GIK solution effects on myocardium exposed to ischemia-reperfusion injury that reduced the reactive oxygen species (155).

More studies need to focus on the diastolic function and explore whether there is a direct relationship between the severity of the acute brain damage and myocardial performance. Because of the pleiotropic cardiovascular manifestations induced by PA, hemodynamic monitoring guided by echocardiographic protocols could be a pivotal point in the management of PA.

As the myocardial function of the neonate is barely adapting to extrauterine conditions, the stress added by inotropic medications is not always the ideal therapeutic procedure. Cardiac dysfunction can be assisted by an extracorporeal device acting as an external blood pump, such as ECMO (extracorporeal membrane oxygenation). As a matter of fact, ECMO is used in neonates in specific conditions as congenital heart disease, myocarditis/cardiomyopathy, arrhythmias, and pulmonary hypertension (including PPHN associated with PA). As opposed to brain injury secondary to PA, cardiac failure *per se* due to PA is not a contraindication for ECMO in neonates. Furthermore, an ECMO-associated brain hemorrhagic event could compromise neurologic outcome (173, 174). Selected cases of neonates with PA, even without PPHN, could thus benefit from this type of circulatory support.

The artificial womb, confirmed as beneficial for premature babies, is another possible solution for allowing the myocardium enough time to adapt to the extrauterine conditions (175). Whether this technique would also be of assistance in term neonates with severe PA, remains a question for further research.

## Conclusions

Despite the recent progresses in perinatal care, the burden of neonatal asphyxia is still not reduced to match the Millennium Developmental Goal (176). The current treatments available rely on magnesium administration, vasopressor support, careful oxygen titration, and, only in well-equipped facilities, hypothermia. The real impact of cardiovascular dysfunction on long-term prognosis of PA survivors is not yet fully understood and evaluated.

Thus, individualized prevention of PA and its cardiovascular effects would be more beneficial than any treatment strategy. This ambitious goal could be achieved through an improved screening system of high-risk pregnancies and close monitoring of fetal warning signs that prompt rapid delivery. Therefore, the moment of early intervention could be shifted even before birth, through prenatal programming, and watchful waiting for early predictive indicators of fetal distress.

## Author Contributions

MP and A-MZ contributed conception and design of this manuscript and wrote the first draft of the manuscript. AP, BP, GP, and LZ wrote sections of the manuscript. All authors contributed to manuscript revision, read, and approved the submitted version.

### Conflict of Interest

The authors declare that the research was conducted in the absence of any commercial or financial relationships that could be construed as a potential conflict of interest.
